# Electrically switched underwater capillary adhesion

**DOI:** 10.1038/s41467-022-32257-5

**Published:** 2022-08-06

**Authors:** Huanxi Zheng, Jing Li, Yongsen Zhou, Chao Zhang, Wanghuai Xu, Yajun Deng, Jiaqian Li, Shile Feng, Zhiran Yi, Xiaofeng Zhou, Xianglin Ji, Peng Shi, Zuankai Wang

**Affiliations:** 1 Department of Mechanical Engineering, City University of Hong Kong, Hong Kong, 999077 China; 2 China-UK Low Carbon College, Shanghai Jiao Tong University, Shanghai, 201306 China; 3 Key Laboratory of Multidimensional Information Processing, School of Communication and Electronic Engineering, East China Normal University, Shanghai, 200241 China; 4 Department of Biomedical Engineering, City University of Hong Kong, Hong Kong, 999077 China; 5 Research Center for Nature-inspired Engineering, City University of Hong Kong, Hong Kong, 999077 China; 6 Shenzhen Research Institute of City University of Hong Kong, Shenzhen, 518057 China

**Keywords:** Engineering, Wetting, Techniques and instrumentation, Surface chemistry

## Abstract

Developing underwater adhesives that can rapidly and reversibly switch the adhesion in wet conditions is important in various industrial and biomedical applications. Despite extensive progresses, the manifestation of underwater adhesion with rapid reversibility remains a big challenge. Here, we report a simple strategy that achieves strong underwater adhesion between two surfaces as well as rapid and reversible detachment in on-demand manner. Our approach leverages on the design of patterned hybrid wettability on surfaces that selectively creates a spatially confined integral air shell to preserve the water bridge in underwater environment. The overall adhesion strength can be multiplied by introducing multiple air shells and rapidly broken by disturbing the integrity of the protective air shell in response to the applied voltage on two surfaces. Our design can be constructed on the flexible substrate with hybrid wettability, which can be applied to non-conductive substrates and adapted to more complicated morphologies, extending the choice of underlying materials.

## Abstract (web summary)

Wang et al. report an underwater capillary adhesive that is strengthened by the conjunction of inner water bridge and outer air shell, and switched timely by a small direct current voltage. The design can also be constructed on flexible tapes, which can be applied to non-conductive substrates.

## Introduction

Reversible underwater adhesion is highly preferred for a high diversity of applications including object transportation^1,2^, underwater robotics^3,4^, and biomedical devices^5,6^. So far, the development of reversible underwater adhesion mainly relies on the synthesis of chemical adhesives by taking inspirations from marine organisms such as mussels, sandcastle worms, and barnacles^7–9^. Generally, these artificial underwater adhesives, in the form of liquid-like glues^10–12^, thin films^13–15^, and bulk (hydro)gels^16–18^, take advantages of bio-inspired chemical groups such as catechol and its derivatives responsible for strong adhesion and stimuli-responsive functionalities for the reversibility. For example, a smart adhesive exhibiting reversible underwater adhesion to different temperatures can be developed by integrating mussel-inspired catechol chemistry with a temperature-responsive polymer through host-guest interactions^19^. Despite extensive progresses, the development of such reversible underwater adhesives remains in infancy, owing to the complicated synthesis of chemical bonds and long response time for the reversibility^19–22^.

In contrast to complicated chemical adhesives, a thin liquid layer trapped between two objects in ambient condition can serve as a simple physical adhesive to achieve strong adhesion. Such a ubiquitous capillary phenomenon has been widely used in nature^23–25^. In this scenario, the Laplace pressure difference between two objects gives rise to an adhesion force that scales as *~ γ* cos*θ*/*d* per unit area, where *γ* and *d* are the surface tension and the thickness of liquid bridge, respectively, and *θ* is the liquid contact angle of the surface^26^. Although the capillary adhesion can be regulated by tuning the surface wettability and flexibility^27–29^, structural geometries^30^ or splitting the liquid bridges^31,32^, achieving efficient underwater adhesion is challenging. First, in the underwater environment, the adherend surface can be infused by the environmental water, which screens the structure effect and prevents the direct contact between the liquid bridge and surfaces. Second, although it has been demonstrated that air bubbles formed between two hydrophobic surfaces can work in underwater environment, the stable adhesion state is easily destroyed at the liquid/gas interface^33–35^. Another challenge emerges in practical implementations where adhesion and detachment in an on-demand manner are highly preferred, both of which, however, pose contradictory requirements on surface design. For example, strong adhesion normally necessitates to preserve a thin and integral capillary bridge, whereas fast detachment needs to destroy the capillary bridge. To this end, developing a capillary surface that enables strong underwater adhesion and rapid responsive detachment still remains challenging.

Herein, we develop a simple underwater capillary adhesive that is strengthened by the conjunction of inner water bridge and outer air shell, and can be also switched timely by the introduction of a small direct current (DC) voltage. On the one hand, the choice of patterned hybrid wettability on adhesive surfaces allows for the selective formation of spatially confined integral air shell, which not only preserves the integrity of water bridge, but also amplifies the pressure difference between the water bridge and water environment for stronger adhesion. On the other hand, the integrity of water bridge and air shell can be destroyed by applying the DC voltage on the adherend surfaces, which triggers the electrolysis inside the water bridge, an electrochemical process leading to the generation of new gas phase. This electrically-switched underwater capillary adhesion can be further applied to flexible materials, providing high adaptability and maneuver to various systems.

## Results and discussion

### Working mechanism

**Fig. 1:**
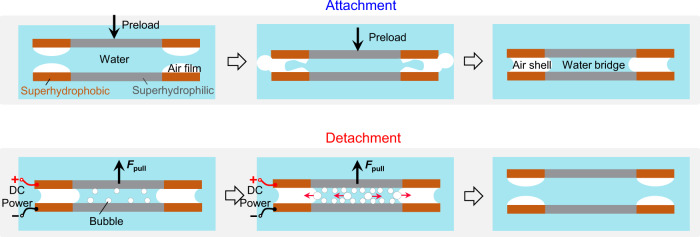
Schematic illustration of the working mechanism for the electrically triggered reversible underwater adhesion. During the attachment process, the formation of spatially confined outer air shell can not only protect the inner water bridge from the water environment, but also enhances the overall underwater adhesion force. While during the detachment process, the electrolysis of water bridge triggered by the DC voltage can disturb the stability of the encapsulated air shell and water bridge, leading to the detachment of the plates.

Figure 1 shows the schematic drawing of our reversible underwater adhesive that mainly leverages on the synergistic cooperation of two core elements: water bridge and air shell. We choose these two elements owing to the strong capillary effect as well as the special feature of water—a gas phase can be generated from liquid phase by applying a small DC voltage through a rapid electrolysis process. We resort to the surface patterned with hybrid wettability to spatially confine a thin water film and an air shell in the preferred location. When being immersed in water, the integral air shell will be confined at the superhydrophobic ring and encapsulate the water bridge trapped at the superhydrophilic circle, leading to strong capillary adhesion. Different from existing adhesives, the capillary adhesive based on the conjunction of water bridge and air shell is reversible, whose adhesion can be rapidly deactivated in an on-demand manner by applying a small voltage, a simple electrolysis process that generates additional gas bubbles to coalescence with the air shell and disturb its integrity.

### Surface design and wettability characterization

**Fig. 2:**
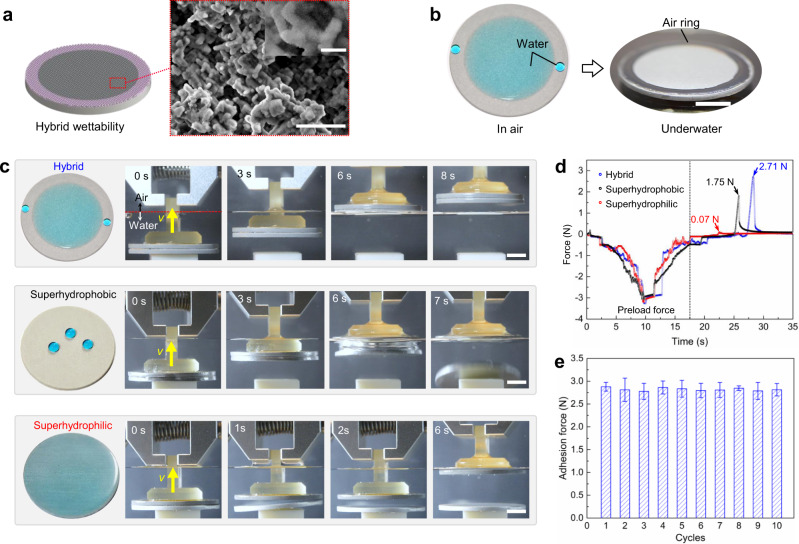
Characterization of underwater capillary adhesion. **a** Schematic drawing and the scanning electron microscopic (SEM) images of the dual-scale structured Al plate with hybrid wettability. The scale bars in the SEM images are both 10 μm. **b** Optical photos of Al plate with hybrid wettability in air and underwater. When being immersed in water, the outer superhydrophobic region of the hybrid Al plate is screened by the uniform air ring, while the inner superhydrophilic region is completely wetted by water. The scale bar is 1 cm. **c** Selected snapshot images comparing underwater adhesion of samples with different surface wettability. Scale bar: 1 cm. **d** The time evolution of the force generated between two plates under a stretching speed of 200 μm s^−1^. The adhesion force of plates with hybrid wettability is ~1.5 and ~39.0 times of that with homogeneous superhydrophobicity and superhydrophilicity, respectively. **e** The cycling test of the encapsulated water bridge-enhanced underwater adhesion, where an average adhesion force of 2.75 N is sustained over 10 cycles. Here, the error bars are the standard deviations of five measurements.

The surface consisting of alternatively patterned superhydrophilic circle surrounded by circular superhydrophobic ring (Fig. 2a) was fabricated based on an Al plate (1060, *Oudifu*). Briefly, as shown in Supplementary Fig. 1a and Methods, an Al plate with a radius (*R*) of 20 mm was first polished by P1500 sandpaper (3 M *Co*.) to remove the oxide layer, followed by electrochemical etching in sodium chloride aqueous solution (molarity: 0.05 M) under a current density of 500 mA cm^−2^ for 5 min to create microscale pits (Supplementary Fig. 1b, c). Later, immersing the cleaned sample in boiling water for 20 min led to the formation of dual-scale structures consisting of microscale pits decorated with nanostructures. Finally, we used the selective fluorination and plasma treatment to render the surface with a hybrid wettability, as exemplified by the water contact angles at the central circle (with a radius ${R}_{{{{{{\rm{SHL}}}}}}}$ of 15 mm) and surrounding ring, being ~2° and ~162°, respectively (see the left panel of Fig. 2b). Supplementary Fig. 2 plots the variation of the wetting contrast as a function of time. On the dual-scale structures, a large wetting contrast >150° is sustained after a long period of over 50 h, while it decays quickly on the sample decorated with single-scale microstructure.

### The performance characterization of underwater capillary adhesion

Moving from air to underwater, the circular superhydrophobic region on the sample is quickly covered by a uniform air ring (see the right panel of Fig. 2b), whereas the central superhydrophilic circle is completely wetted by water. By aligning two samples and applying force to drain out the excess water at the center, a thin water bridge and an integral air shell can be formed (Supplementary Fig. 3), in which the air shell encapsulates and preserves the thin water bridge from the water environment. The formation of the air shell and water bridge is evidenced by our visualization as shown in Supplementary Fig. 4, in which a glass plate with hybrid wettability is chosen as the top plate (see Methods).

We next measured the adhesion force between two hybrid Al plates using a home-made device (Methods and Supplementary Fig. 5). As shown in the selected images in Fig. 2c and Supplementary Movie 1, two hybrid Al plates attach tightly together underwater, and such an attachment is maintained even above water. In contrast, the adhesion enabled by homogeneous superhydrophobicity collapses when the plates are pulled out of the water. And there is no noticeable underwater adhesion between two superhydrophilic plates, suggesting the importance of air shell in maintaining the capillary adhesion. Figure 2d plots the variation of the measured adhesion forces for different samples, in which the top plate is vertically lifted under a constant speed of 200 μm s^−1^ and the bottom plate is fixed by a gripper. The adhesion force rendered by the adhesive with hybrid wettability is measured at ~2.7 N, which is ~1.5 and ~39.0-folds of that with homogeneous superhydrophobicity and superhydrophilicity, respectively. In addition to the surface wettability, the preload also plays an important role in the underwater adhesion. As shown in Supplementary Fig. 6, at the beginning, the adhesion force rises as the preload force increases, indicating a decrease in the thickness of water bridge and air shell. And there exists a threshold preload force of 3 N, beyond which the adhesion force is stabilized at ~2.75 N, suggesting that the thickness of water bridge and air shell (*h*) reaches the roughness scale of the structure (i.e., $h \; \approx$ 95 μm). More importantly, our underwater capillary adhesive is reusable and highly durable. As evidenced by Fig. 2e and Supplementary Fig. 7, the capillary adhesive is sustained over 10 testing cycles, and can hold a load of 100 g in the underwater environment for more than 48 h.

### Underwater adhesion force analysis and amplification

**Fig. 3:**
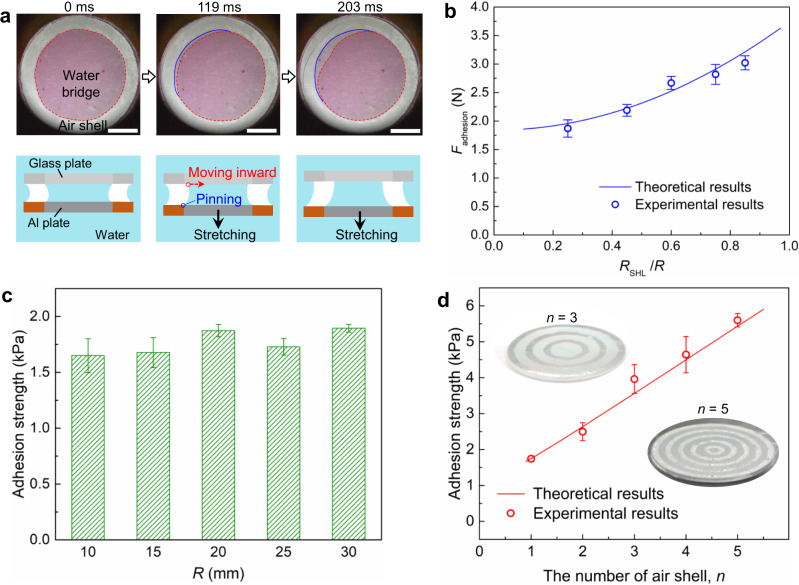
Adhesion force analysis and amplification. **a** The selected snapshots and the schematic images showing the dynamics of air shell and water bridge during the stretching. Here, the hybrid transparent glass plate is set as the top visualization window. The dark red region with the dotted line and the light red region with the solid line are the contact areas of water bridge on the top glass plate and bottom Al plate, respectively. Here, the scale bar is 1 cm. **b** The variation of theoretical and experimental adhesion force as a function of *R*_SHL_/*R*. Our theoretical results show good agreement with the experimental measurements. **c** The variation of adhesion strength as a function of sample size *R*. When we kept *R*_SHL_/*R* at 0.5 and increase the *R*, the adhesion strength can be stabilized at ~1.75 kPa, suggesting the scalability of our design. **d** The variation of the theoretical and experimental adhesion forces as a function of air shell number. The error bars in **b**–**d** denote the standard deviations of five measurements.

How does the integration of the outer air shell and inner water bridge elevate the performance of underwater capillary adhesive? To answer this question, we first characterized the dynamics of the interface between air shell and water bridge during stretching. We colored the inner water bridge using methyl red, and pressed the transparent and hybrid glass plate at the top using a preload of 3 N. Figure 3a shows the time-lapsed optical images and the corresponding schematics revealing the motions of water bridge, with the dark red region (the dotted red line) and the light red region (the solid blue line) indicating the contact areas of water bridge on the top glass plate and bottom Al plate, respectively. Upon stretching of Al plate, the top contact line of water bridge recoils owing to higher receding contact angle of glass plate (i.e., 24.3°), pulling the liquid/air interface inward (Supplementary Movie 2). In contrast, the bottom contact line of water bridge is completely pinned at the superhydrophobic-superhydrophilic junction of the hybrid Al plate. Thus, between two hybrid Al plates, a constant plate distance of *h* and a fixed contact radius of water bridge, ${R}_{{{{{{\rm{SHL}}}}}}}$, are expected, considering ${R}_{{{{{{\rm{SHL}}}}}}}\gg \; h$ and the incompressibility of water.

We next developed a theoretical model to predict the underwater adhesion force (Supplementary Fig. 8). Note that owing to the superwettability of the hybrid surface, the surface tension force at the liquid/air interface can be ignored. Thus, the adhesion component generated by the air shell can be calculated as (see Methods)^26,33^: ${F}_{{{{{{\rm{a}}}}}}}=\triangle {P}_{{{{{{\rm{a}}}}}}}{A}_{{{{{{\rm{a}}}}}}}=2\pi [{R}^{2}-{R}_{{{{{{\rm{SHL}}}}}}}^{2}]\gamma \, {{\cos }}\left(\pi -{\theta }_{{{{{{\rm{SHB}}}}}},{{{{{\rm{a}}}}}}}\right)/h$, where $\triangle {P}_{{{{{{\rm{a}}}}}}}$ is the difference between the pressure of air shell and the hydrostatic pressure of water environment, ${A}_{{{{{{\rm{a}}}}}}}$ is the contact area of air shell, ${\theta }_{{{{{{\rm{SHB}}}}}},{{{{{\rm{a}}}}}}}=$165°, represents the advancing contact angle of water on the outer superhydrophobic region. Meanwhile, the pressure difference between the inner water bridge and the water environment can be obtained by multiplying the Laplace pressures at the outer and inner water/air interfaces as: $\triangle {P}_{{{{{{\rm{l}}}}}}}=2\gamma \left[{{\cos }}\; {\theta }_{{{{{{\rm{SHL}}}}}},{{{{{\rm{r}}}}}}}+\,{{\cos }}\; \left(\pi -{\theta }_{{{{{{\rm{SHB}}}}}},{{{{{\rm{a}}}}}}}\right)\right]/h$. As a result, the adhesion component arising from the water bridge can be amplified as ${F}_{{{{{{\rm{l}}}}}}}=\triangle {P}_{{{{{{\rm{l}}}}}}}{A}_{{{{{{\rm{l}}}}}}}=2\pi {R}_{{{{{{\rm{SHL}}}}}}}^{2}\gamma \left[{{\cos }}\; {\theta }_{{{{{{\rm{SHL}}}}}},{{{{{\rm{r}}}}}}}+{{\cos }}\; \left(\pi -{\theta }_{{{{{{\rm{SHB}}}}}},{{{{{\rm{a}}}}}}}\right)\right]/h$, where ${A}_{{{{{{\rm{l}}}}}}}$ is the contact area of water bridge, ${\theta }_{{{{{{\rm{SHL}}}}}},{{{{{\rm{r}}}}}}}$ ≈ 0°, denotes the receding contact angle of water on the central superhydrophlic region. Thus, the overall underwater adhesion can be obtained as:

1 $${F}_{{{{{{\rm{ad}}}}}}{{{{{\rm{h}}}}}}{{{{{\rm{esion}}}}}}}=\,{F}_{{{{{{\rm{a}}}}}}}+{F}_{{{{{{\rm{l}}}}}}}=\frac{2\pi \gamma {R}^{2}}{h}\left[{\left(\frac{{R}_{{{{{{\rm{SHL}}}}}}}}{R}\right)}^{2}{{\cos }}\; {\theta }_{{{{{{\rm{SHL}}}}}},{{{{{\rm{r}}}}}}}-{{\cos }}\; {\theta }_{{{{{{\rm{SHB}}}}}},{{{{{\rm{a}}}}}}}\right]$$

Based on the equation, the underwater adhesion increases parabolically as ${R}_{{{{{{\rm{SHL}}}}}}}/R$, which is in good agreement with the experimental results (see Fig. 3b). Moreover, as evidenced by Fig. 3c, by keeping the area ratio of the superhydrophilic region (i.e., ${R}_{{{{{{\rm{SHL}}}}}}}/R$) the same, the underwater capillary strength (${F}_{{{{{{\rm{ad}}}}}}{{{{{\rm{h}}}}}}{{{{{\rm{esion}}}}}}}/\pi {R}^{2}$) is kept constant when increasing *R*, suggesting the scalability of our capillary adhesive.

Based on the above analysis, the occurrence of the outer air shell can promote the adhesion force of the inner capillary bridge. Thus, we hypothesize that by increasing the number of air shell, the overall capillary strength can be further enhanced. To verify such a hypothesis, we consider *n* air shells with width of $R/2n$ that are distributed evenly on the plate. As schematically shown in Supplementary Fig. 9, upon stretching, both the front (red line) and rear menisci (yellow line) of ring-shaped water bridge move towards the center of the plates, during which the dynamic contact angles are the advancing contact angle of the superhydrophilic region and the receding contact angle of superhydrophobic region, respectively. The adjacent air shells and water bridges form pressure relays, leading to larger pressure difference between the inner water bridge/air shell and the water environment (see Methods). Accordingly, the overall underwater capillary adhesion strength can be calculated as:

2 $$\frac{{F}_{{{{{{\rm{n}}}}}}}}{\pi {R}^{2}}=\frac{2\gamma }{h}\left[-\left(\frac{3n+1+2{n}^{2}}{6n}\right){{\cos }}\; {\theta }_{{{{{{\rm{SHB}}}}}},{{{{{\rm{a}}}}}}}+\left(\frac{4{n}^{2}-1}{12n}\right){{\cos }}\; {\theta }_{{{{{{\rm{SHL}}}}}},{{{{{\rm{r}}}}}}}\right]$$

Figure 3d shows the variation of underwater adhesion strength as a function of *n*. Based on the plot, the overall adhesion strength under *n* = 5 is 5.43 kPa, which is 3.1 times of that under single air shell. Notably, without the need of synthesis of complicated chemical materials, the underwater adhesion strength can be elevated by further improving the number of air shell *n*. For example, a large adhesion strength of ~95 kPa is expected under an air shell number of 100, and such an adhesion strength can reach ~472 kPa when air shell number is increased to 500, which is comparable with the state-of-the-art adhesives^2,19–22,36–45^ (Supplementary Fig. 10).

### Reversible underwater capillary adhesion triggered by electricity

**Fig. 4:**
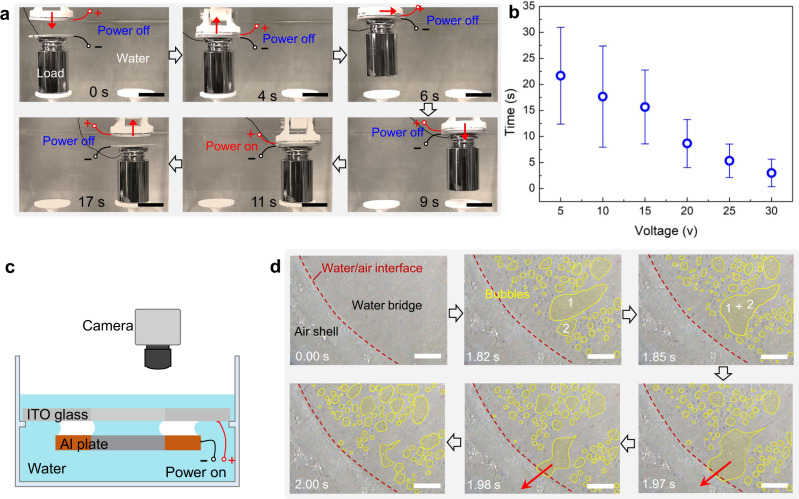
On-demand reversible underwater adhesion triggered by electricity. **a** Selected snapshot images showing the fast and on-demand pick-up and release of 200 × *g* metal load under a DC voltage of 20 V. Here, the scale bar is 2 cm. **b** The response time of reversible underwater adhesion is regulated by the applied voltage. The error bars are the standard deviations of five measurements. **c** The schematic diagram of the experimental setup to visualize the electrolysis process inside the water bridge. **d** Selected snapshots showing the generation and movement of bubbles during the electrolysis of water in the water bridge. Scale bar: 2 mm.

More intriguingly, the underwater capillary adhesion can be switched rapidly by applying a small voltage, enabling the picking-up and release of objects underwater in an on-demand manner. As shown in Fig. 4a and Supplementary Movie 3, under a DC power voltage of 20 V, a metal load of 200 g can be moved to any pre-designed locations, and released within a short time of 6 s. Here, the number and width of air shell are set at 3 and 6.5 mm, respectively. The response time for the controlled object release can be further regulated by adjusting the applied voltage. As shown in Fig. 4b, the response time drops to 3 s when the voltage is increased to 30 V, which is much shorter than conventional underwater reversible adhesives that rely on thermal^2^ or light^19^ stimuli. We further explored the fundamental mechanism of such an electrically triggered on-demand reversible underwater adhesion using the setup in Fig. 4c. Here, the ITO-coated transparent glass with hybrid wettability was settled as the visualization window. As shown in the selected snapshot images in Fig. 4d and Supplementary Movie 4, the introduction of a DC voltage of 20 V triggers the onset of electrolysis within the water bridge. On the one hand, the generation of extensive gas bubbles inside the water bridge decreases the contact area between the plates and water bridge, and impairs the adhesion force of water bridge. On the other hand, the continuous growth of these bubbles leads to their connection with the outer air shell, which increases its pressure and thus decreases the adhesion force. Finally, when the overall adhesion force resulting from the conjunction of water bridge and air shell becomes less than the weight of load, both the water bridge and air shell collapse.

**Fig. 5:**
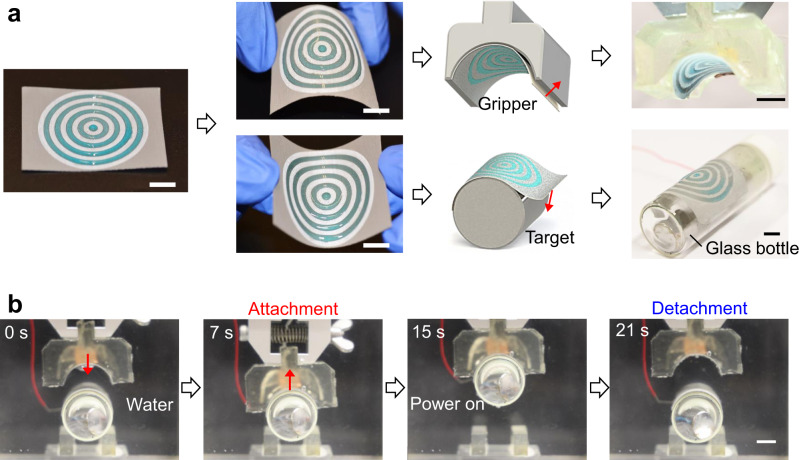
The flexible underwater capillary adhesive. **a** The construction of flexible underwater capillary adhesives on commercial Al tape that is treated with hybrid wettability. Here, the scale bar is 1 cm and the width of air ring is set at 2 mm. **b** Selected snapshots showing the on-demand pick-up and release of non-conductive glass cylinder by applying the flexible adhesives between the glass cylinder and its counterpart. Scale bar: 1 cm.

Finally, we demonstrate the construction of reversible underwater capillary adhesive on commercial and flexible Al tape (BenYiDa Company) with a thickness of 150 μm (see Fig. 5a). The top surface of Al tape is treated with hybrid wettability and the air shell number is set at 5. The flexible adhesives can be closely attached to both convex and concave surfaces as shown in Fig. 5b and Supplementary Movie 5, making it possible to exhibit the on-demand pick-up and release of object, otherwise impossible achieved by the commercial tape underwater. In a border perspective, the flexible adhesive can be applied between a pair of adherents with different morphologies and electrical conductivities, which will find applications in the underwater detection and locomotion of smart robotics.

## Methods

### Fabrication of dual-scale structured Al surface with hybrid wettability

An Al sheet (1060) with a radius of 20 mm was first polished by abrasive papers to remove the oxide layer. Then, the polished sheet was electrochemically etched in 0.05 M sodium chloride solution with the current density and etching time being 500 mA·cm^−2^ and 5 min, respectively. The etched sheet was ultrasonically cleaned in deionized water and then immersed into boiling deionized water for 20 min. After drying, the sheet was immersed in a trichloro(1H,1H,2H,2H-perfluorooctyl)silane *n*-hexane solution (*AQA*) with a mass fraction of 0.001 for 15 min followed by 10 min heating in thermostat with a temperature of 130 °C. Assisted by a hollow mask with an inner radius of 15 mm and an outer radius of 20 mm, the central region was treated to be superhydrophilic by Plasma Cleaner PDC-32G (Harrick Plasma limited.). After removing the mask, we obtained the dual-scale structured surface with hybrid wettability.

### Fabrication of transparent glass plate with hybrid wettability

A glass plate with a radius of 20 mm was cleaned by alcohol and dried in a thermostat. After 5 min of plasma treatment, the NeverWet Multi-Surface (*Rust Oleum*) superhydrophobic spray was alternatively applied to the surrounding region of the surface with a width of 5 mm using a patterned mask. Finally, the transparent glass plate was patterned with a hybrid wettability, where the contact angles at central and surrounding regions were measured at 32.3° (advancing angle: 60.5°, receding angle: 24.3°) and 161.1° (advancing angle: 165.0°, receding angle: 139.5°), respectively.

### Construction of the force measurement device

The force measurements were carried out by a home-made device shown in Supplementary Fig. 5, which consists of a vertical lifting system, force sensor, force preload component, leveling device and sample grippers. During the measurements, the top sample was lift up vertically under a speed of 200 μm s^−1^, while the bottom sample was fixed by a gripper. The adhesion force generated by the encapsulated water bridge was measured by the force sensor, which was connected to the computer with data acquisition card.

### Modeling of the underwater adhesion strength of multiple air shells

Under the air shell number of *n*, the overall adhesion force can be calculated as:

1 $${F}_{{{{{{\rm{n}}}}}}}={F}_{{{{{{\rm{a}}}}}},1}+{F}_{{{{{{\rm{l}}}}}},1}+{F}_{{{{{{\rm{a}}}}}},2}+{F}_{{{{{{\rm{l}}}}}},2}+\cdots {F}_{{{{{{\rm{a}}}}}},{{{{{\rm{n}}}}}}}+{F}_{{{{{{\rm{l}}}}}},{{{{{\rm{n}}}}}}}$$

Here, ${F}_{{{{{{\rm{a}}}}}}}$ and ${F}_{{{{{{\rm{l}}}}}}}$ are the adhesion forces of air shell and water bridge, respectively. As schematically shown in Supplementary Fig. 9, in the underwater environment, the inner and outer menisci of the ring-shaped water bridge both move towards the center of the plates. Thus, the dynamic contact angles at the inner and outer contact lines are determined by the advancing contact angle of superhydrophobic region and the receding contact angle of superhydrophilic region, respectively. In this way, the adhesion force of the inner water bridges and air shells can be obtained by multiplying the Laplace pressures generated at the outer air/water interfaces. For example, the adhesion forces of air shell 1 and water bridge 1 can be obtained as:

2 $${F}_{{{{{{\rm{a}}}}}},1}=-\frac{2\pi \gamma }{h}\left\{{R}^{2}-{\left[\frac{\left(2n-1\right)R}{2n}\right]}^{2}\right\}\,{{\cos }}\; {\theta }_{{{{{{\rm{SHB}}}}}},{{{{{\rm{a}}}}}}}$$

3 $${F}_{{{{{{\rm{l}}}}}},1}=\frac{2\pi \gamma }{h}\left\{{\left[\frac{\left(2n-1\right)R}{2n}\right]}^{2}-{\left[\frac{\left(2n-2\right)R}{2n}\right]}^{2}\right\}\left(-{{\cos }}\; {\theta }_{{{{{{\rm{SHB}}}}}},{{{{{\rm{a}}}}}}}+\,{{\cos }}\; {\theta }_{{{{{{\rm{SHL}}}}}},{{{{{\rm{r}}}}}}}\right)$$

Similarly, for air shell 2 and water bridge 2:

4 $${F}_{{{{{{\rm{a}}}}}},2}=\frac{2\pi \gamma }{h}\left\{{\left[\frac{\left(2n-2\right)R}{2n}\right]}^{2}-{\left[\frac{\left(2n-3\right)R}{2n}\right]}^{2}\right\}\left(-2{{\cos }}\; {\theta }_{{{{{{\rm{SHB}}}}}},{{{{{\rm{a}}}}}}}+\,{{\cos }}\; {\theta }_{{{{{{\rm{SHL}}}}}},{{{{{\rm{r}}}}}}}\right)$$

5 $${F}_{{{{{{\rm{l}}}}}},2}=\frac{2\pi \gamma }{h}\left\{{\left[\frac{\left(2n-2\right)R}{2n}\right]}^{2}-{\left[\frac{\left(2n-3\right)R}{2n}\right]}^{2}\right\}\left(-2{{\cos }}\; {\theta }_{{{{{{\rm{SHB}}}}}},{{{{{\rm{a}}}}}}}+2{{\cos }}\; {\theta }_{{{{{{\rm{SHL}}}}}},{{{{{\rm{r}}}}}}}\right)$$

For air shell *i* and water bridge *i*, we have:

6 $${F}_{{{{{{\rm{a}}}}}},{{{{{\rm{i}}}}}}}=\frac{2\pi \gamma {R}^{2}}{h}\left\{-\left[\frac{4n-\left(4i-3\right)}{4{n}^{2}}i\right]{{\cos }}\; {\theta }_{{{{{{\rm{SHB}}}}}},{{{{{\rm{a}}}}}}}+\left[\frac{4n-\left(4i-3\right)}{4{n}^{2}}\left(i-1\right)\right]{{\cos }}\; {\theta }_{{{{{{\rm{SHL}}}}}},{{{{{\rm{r}}}}}}}\right\}$$

7 $${F}_{{{{{{\rm{l}}}}}},{{{{{\rm{i}}}}}}}=\frac{2\pi \gamma {R}^{2}}{h}\left\{-\left[\frac{4n-\left(4i-1\right)}{4{n}^{2}}i\right]{{\cos }}\; {\theta }_{{{{{{\rm{SHB}}}}}},{{{{{\rm{a}}}}}}}+\left[\frac{4n-\left(4i-1\right)}{4{n}^{2}}i\right]{{\cos }}\; {\theta }_{{{{{{\rm{SHL}}}}}},{{{{{\rm{r}}}}}}}\right\}$$

Thus, the overall underwater adhesion strength can be calculated as:

8 $$\frac{{F}_{{{{{{\rm{n}}}}}}}}{\pi {R}^{2}}=\frac{\mathop{\sum }\nolimits_{{{{{{\rm{i}}}}}}=1}^{{{{{{\rm{n}}}}}}}{F}_{{{{{{\rm{a}}}}}},{{{{{\rm{i}}}}}}}+\mathop{\sum }\nolimits_{{{{{{\rm{i}}}}}}=1}^{{{{{{\rm{n}}}}}}}{F}_{{{{{{\rm{l}}}}}},{{{{{\rm{i}}}}}}}}{\pi {R}^{2}}=\frac{2\gamma }{h}\left[-\left(\frac{3n+1+2{n}^{2}}{6n}\right){{\cos }}\; {\theta }_{{{{{{\rm{SHB}}}}}},{{{{{\rm{a}}}}}}}+\left(\frac{4{n}^{2}-1}{12n}\right){{\cos }}\; {\theta }_{{{{{{\rm{SHL}}}}}},{{{{{\rm{r}}}}}}}\right]$$

Based on the model, the overall adhesion strength can be promoted greatly by increasing the number of air shell *n*.

### The visualization of the electrolysis inside the water bridge

To visualize the electrolysis inside the water bridge, a piece of commercial ITO glass slide of 80 mm × 50 mm × 1.5 mm was used to replace the upper Al sample. The ITO surface was selectively patterned with a hybrid wettability by selectively applying the NeverWet Multi-Surface spray. Then, two wires were connected to the ITO and Al samples using conductive copper tape. A high-speed camera was fixed above the device to capture the dynamic electrolysis process of water from the top view.

### Reporting summary

Further information on research design is available in the Nature Research Reporting Summary linked to this article.

## Supplementary information

Supplementary Information

Description of Additional Supplementary Files

Supplementary Movie 1

Supplementary Movie 2

Supplementary Movie 3

Supplementary Movie 4

Supplementary Movie 5

Reporting Summary

## Data Availability

The data that support the findings of this study are available from the corresponding authors on reasonable request.
