# Supplementary material for: Electrically switched underwater capillary adhesion
Source: Nat Commun. 2022 Aug 6;13:4584. doi: 10.1038/s41467-022-32257-5 (PMC9357018; doi:10.1038/s41467-022-32257-5)
Supplement: Supplementary file 1 — Supplementary Information [file 41467_2022_32257_MOESM1_ESM.pdf]

Supplementary information

Electrically-switched underwater capillary adhesion

Huanxi Zheng[1#], Jing Li[1,2#], Yongsen Zhou[1], Chao Zhang[1], Wanghuai Xu[1], Yajun Deng[1], Jiaqian Li[1], Shile Feng[1], Zhiran Yi[1], Xiaofeng Zhou[3], Xianglin Ji[4], Peng Shi[4], Zuankai Wang[1,5,6*]

[1]Department of Mechanical Engineering, City University of Hong Kong, Hong Kong 999077, China

[2]China-UK Low Carbon College, Shanghai Jiao Tong University, Shanghai 201306, China

[3]Key Laboratory of Multidimensional Information Processing, School of Communication and Electronic Engineering, East China Normal University, Shanghai 200241, China

[4]Department of Biomedical Engineering, City University of Hong Kong, Hong Kong 999077, China

[5]Research Center for Nature-inspired Engineering, City University of Hong Kong, Hong Kong 999077, China

[6]Shenzhen Research Institute of City University of Hong Kong, Shenzhen 518057, China

#These authors contribute equally to this manuscript

*Corresponding author: zuanwang@cityu.edu.hk (Z.W.).

Supplementary Figures

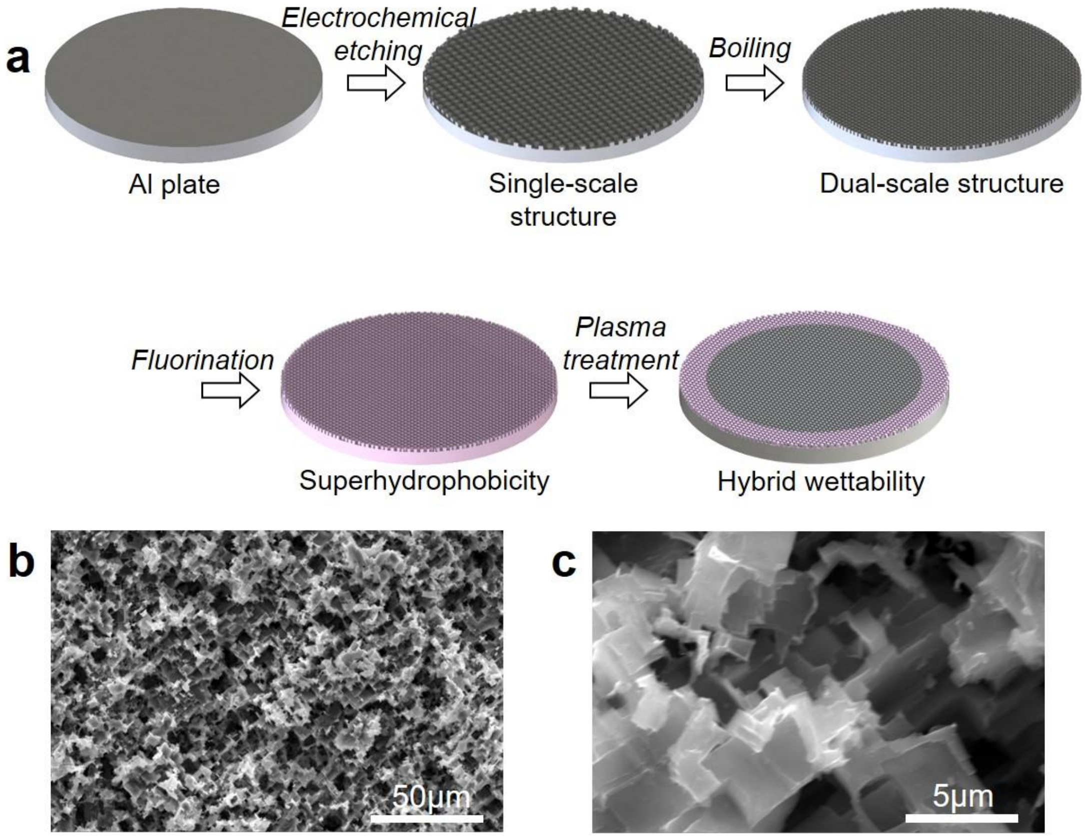

Supplementary Fig. 1. The fabrication and characterization of hybrid samples. a. Schematic drawing of the fabrication processes of dual-scale structured Al plate with hybrid wettability. **(b**, **c)** The SEM images showing the Al surfaces decorated with single-scale micropits.

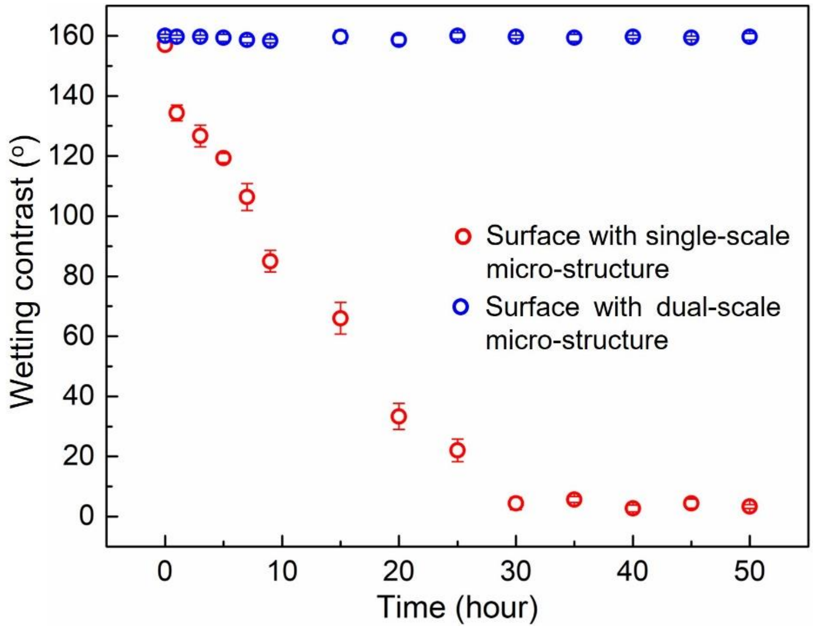

Supplementary Fig. 2. Time evolution of the wetting contrast on surfaces with hybrid wettability. On the dual-scale structured surface, a large wetting contrast > 150° can be sustained over a long period of 50 h, while on the single-scale structured surface, it decays quickly as time proceeds. The error bars are the standard deviations of five measurements.

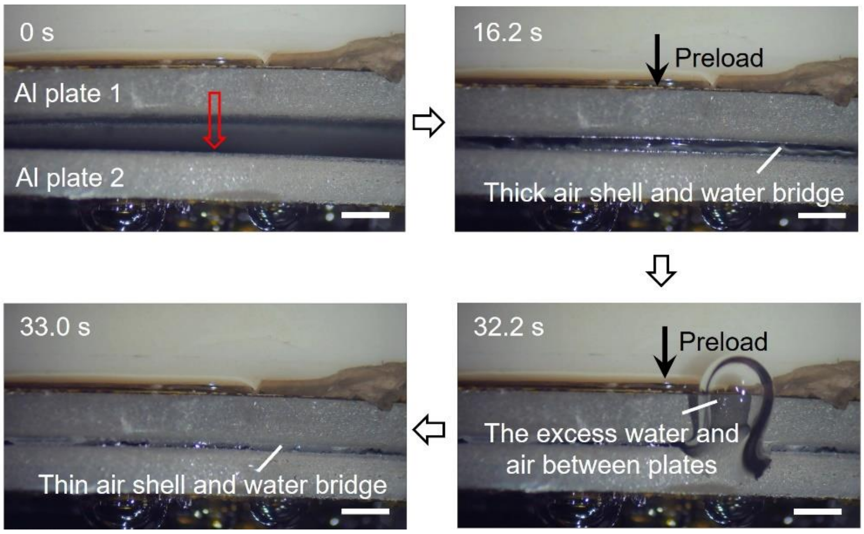

Supplementary Fig. 3. Selected snapshots showing the attachment of two hybrid Al plates under a preload force of 3 N. By applying a preload of 3 N to the top plate, the excess water and air trapped between plates are squeezed out, forming thin water bridge and air shell that seal the two plates tightly together. Here, the scale bar is 2 mm.

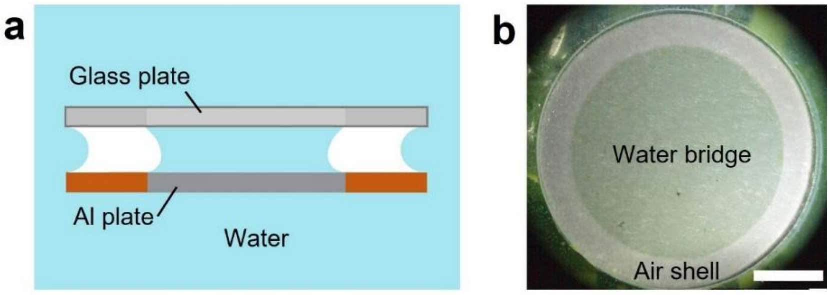

Supplementary Fig. 4. Visualization of the protective air shell and encapsulated water bridge formed between two hybrid plates. a, Schematic drawing of the experimental setup, where the transparent and hybrid glass plate is set as the top visualization window. **b**, Optical photo demonstrating the formation of integral air shell and water bridge. The scale bar is 1 cm.

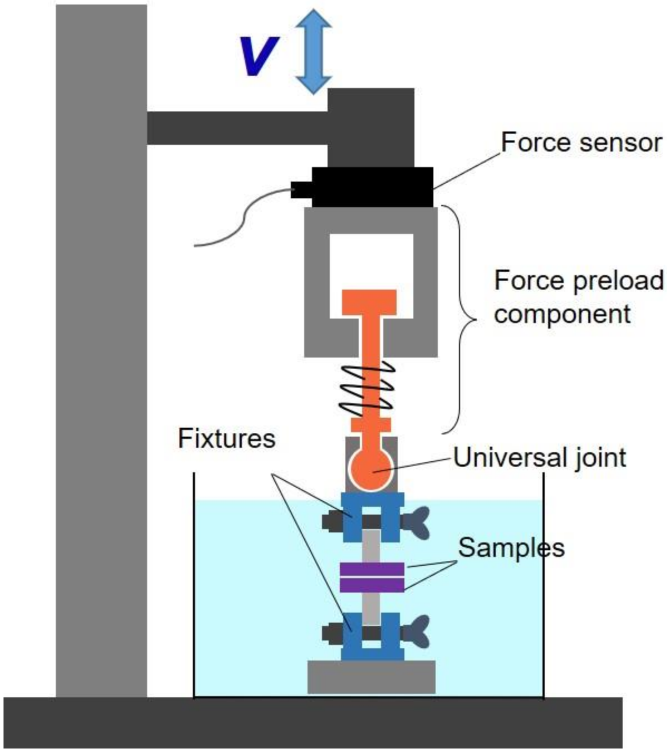

Supplementary Fig. 5. Home-made device for the measurement of the underwater adhesion. The device consists of vertical lifting system, force sensor, force preload component, leveling device and samples gripper. During the measurements, the top plate was lift up vertically under a speed of 200 μm/s, while the bottom sample is fixed by a gripper.

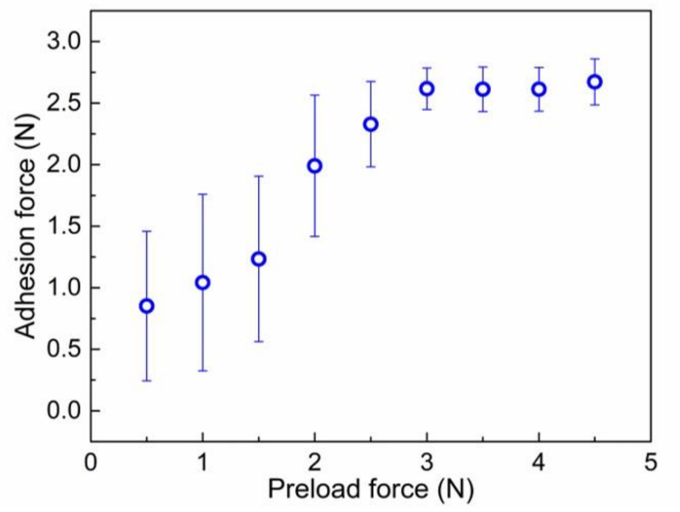

Supplementary Fig. 6. The variation of adhesion force as a function of preload force. When the preload force is larger than 3 N, the adhesion force is stabilized at ~2.75 N, suggesting that the thickness of water bridge is decreased to the height of the dual-scale structure of the Al plate. Here, the error bars denote the standard deviations of five measurements

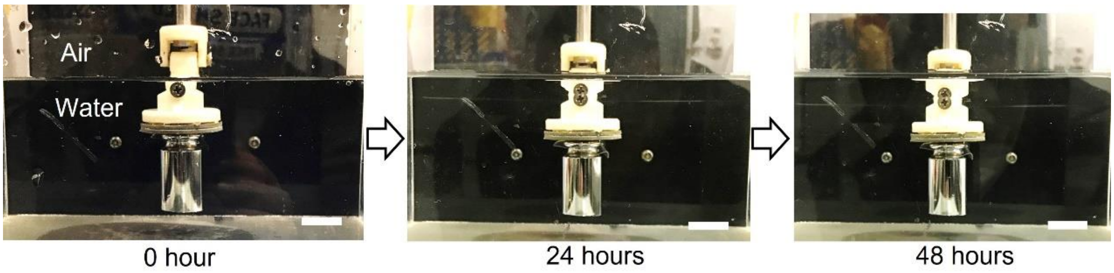

Supplementary Fig. 7. Optical images proving the sustainability of the underwater capillary adhesive. The capillary adhesive can hold a load of 100 g underwater for more than 48 hours. Here, the scale bar is 2 cm.

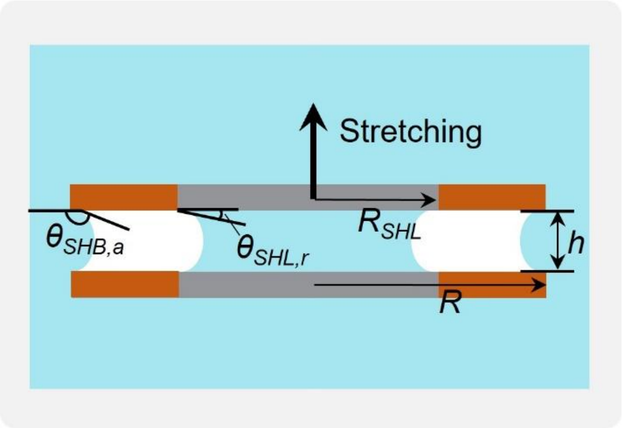

Supplementary Fig. 8. Schematic image showing the dynamics of air/water interfaces. During the stretching, the dynamic contact angles of outer and inner air/water interface are determined by the advancing contact angle of superhydrophobic region ($\theta_{\mathrm{SHB,a}}$) and the receding contact angle of superhydrohobilic region ($\theta_{\mathrm{SHL,r}}$), respectively.

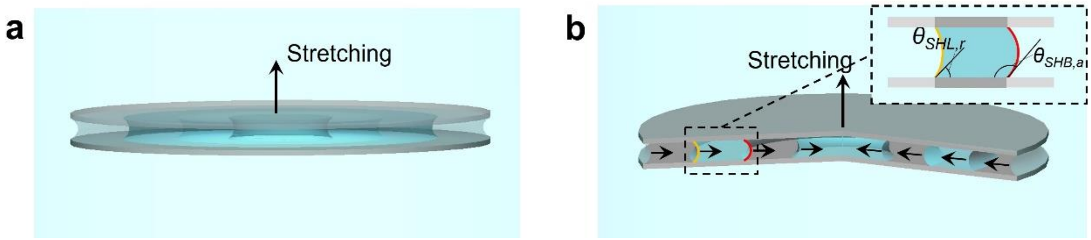

Supplementary Fig. 9. The schematic images showing the side view (a) and cross section (b) of multiple water bridges and protective air shells. Upon stretching, the front (red line) and rear (yellow line) menisci of water bridge both move towards the center of the plates, leading to the convex and concave geometries of the front and rear meniscus, respectively.

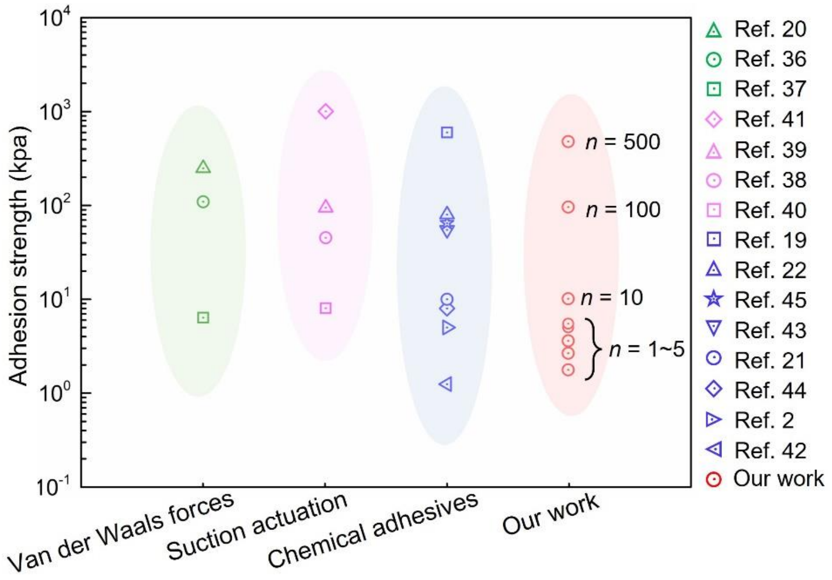

Supplementary Fig. 10. The comparison of the adhesion strength between our work and the state-of-the-art adhesives. Note that without the need of the synthesis of complicated chemical materials, the underwater adhesion strength of our design can be enhanced greatly by introducing multiple air shells. For example, under an air shell number of 500, the adhesion strength can reach ~472 kPa, which is comparable to the state-of-the-art adhesives.
