# Supplementary material for: Electrically switched underwater capillary adhesion
Source: Nat Commun. 2022 Aug 6;13:4584. doi: 10.1038/s41467-022-32257-5 (PMC9357018; doi:10.1038/s41467-022-32257-5)
Supplement: Supplementary file 2 — Description of Additional Supplementary Files [file 41467_2022_32257_MOESM2_ESM.pdf]

Description of Additional Supplementary Files

File Name: Supplementary Movie 1
Description: The underwater adhesion performance of samples with different surface wettability. The capillary adhesion generated by hybrid Al plates works both underwater and above water. In contrast, the adhesion enabled by homogeneous superhydrophobicity collapses when the plates are pulled out of the water. And there is no noticeable underwater adhesion between two superhydrophilic plates.

File Name: Supplementary Movie 2
Description: The dynamics of water/air interfaces during stretching. To visualize the water/air interfaces, we colored the inner water bridge using methyl red, and pressed the transparent hybrid glass plate at the top using a preload of 3 N. We observed a completely pinned contact line of water bridge on the bottom hybrid Al plate during stretching.

File Name: Supplementary Movie 3
Description: The electrically-triggered fast and on-demand pick-up and release of 200 g metal load. Here, the DC voltage is set at 20 V.

File Name: Supplementary Movie 4
Description: The generation and growth of bubbles caused by the electrolysis of water bridge. The gas bubbles not only decrease the contact aera of inner water bridge, but also increase the pressure of outer air shell as they leak to the air shell, both of which lead to the collapse of underwater adhesion.

File Name: Supplementary Movie 5
Description: The on-demand pick up and release of non-conductive glass cylinder. By applying the flexible adhesives between the glass cylinder and its counterpart, the reversible adhesion that is impossible on commercial tapes in underwater environment can be achieved.
